# Developing a natural language processing system using transformer-based models for adverse drug event detection in electronic health records

**DOI:** 10.1371/journal.pone.0350516

**Published:** 2026-07-01

**Authors:** Jingyuan Wu, Xiaodi Ruan, Elizabeth McNeer, Katelyn M. Rossow, Leena Choi

**Affiliations:** 1 Data Science Institute, Vanderbilt University, Nashville, Tennessee, United States of America; 2 Department of Psychology and Human Development, Vanderbilt University, Nashville, Tennessee, United States of America; 3 Department of Biostatistics, Vanderbilt University Medical Center, Nashville, Tennessee, United States of America; 4 Department of Pediatrics, Vanderbilt University Medical Center, Nashville, Tennessee, United States of America; Centro de Investigacion en Ciencias de Informacion Geoespacial AC (Research Center on Geospatial Information Sciences), MEXICO

## Abstract

**Objective:**

To develop a transformer-based natural language processing (NLP) system for detecting adverse drug events (ADEs) from clinical notes in electronic health records (EHRs).

**Materials and Methods:**

We fine-tuned BERT Short-Formers and Clinical-Longformer using the processed dataset from the 2018 National NLP Clinical Challenges (n2c2) shared task Track 2. Two data processing methods, window-based and split-based approaches, were compared to identify the optimal processing method. Model generalizability was evaluated on a dataset extracted from Vanderbilt University Medical Center (VUMC) EHRs.

**Results:**

On the n2c2 dataset, the best 5-fold cross-validation AUPRC, micro F1, and macro F1 scores were 0.840 (Clinical-Longformer, 4-chunk split), 0.965 (BioBERT, 15-word window), and 0.852 (Clinical-Longformer, 10-chunk split). On the VUMC dataset, the best AUPRC, micro F1, and macro F1 scores were 0.536 (Clinical-Longformer, 6-chunk split), 0.966 (BERT-base-uncased, 6-chunk split), and 0.762 (Clinical-Longformer, 4-chunk split).

**Discussion:**

Transformer-based models demonstrated strong performance for ADE detection, with split-based processing generally outperforming window-based methods. Clinical-Longformer combined with a practical split-based approach showed promise for real-world implementation. Beyond token limits, chunk size substantially influenced model performance, even when text length remained within limits.

**Conclusion:**

Our findings provide practical guidance for developing transformer-based ADE detection systems from clinical notes. The selection of both text preprocessing strategies and model architectures should be guided by note characteristics and practical considerations such as annotation burden.

## Background and significance

Adverse drug events (ADEs) refer to any physical or psychological injuries and unexpected events caused by medication use [1], and can be significant hospital complications impacting patient experience and costs [2]. It is challenging to detect ADEs at an early stage due to ambiguous and incomprehensive information about actions, symptoms and medications. Thus, information extracted from electronic health records (EHRs), including diagnoses, clinical notes, and laboratory tests, becomes important for supporting early treatment and primary and secondary prevention.

However, extracting relevant information from unstructured clinical notes is challenging. Over the past few decades, several approaches have been used including rule-based, machine learning-based, deep learning-based, and contextualized language model-based approaches [3]. More recently, contextualized language models have been widely applied in natural language processing (NLP). These models are pre-trained on large corpora and can effectively capture contextual patterns in the relevant domain, thereby facilitating their application in downstream tasks. The Bidirectional Encoder Representations from Transformer (BERT) model [4,5], one of the most widely adopted contextualized models, was first introduced in 2018. It employs a transformer architecture to pre-train a deep neural network on a large corpus of unlabeled text data using an unsupervised approach. Since BERT has continuously evolved, BERT-based NLP systems have shown great promise for ADE-related tasks, such as relation extraction and text classification [3,6]. Fan *et al.* [7] conducted a comparative analysis between BERT-based models, standard deep learning models, and current state-of-the-art models for ADE detection and extraction, and demonstrated that a BERT-based model achieved new state-of-the-art results. Hussain *et al.* [8] proposed an end-to-end system for adverse drug relation detection by fine-tuning BERT, showing good performance on Twitter and biomedical literature datasets (e.g., PubMed-indexed abstracts). The introduction of the SpanBERT (Span-based BERT) architecture [9] for ADE extraction task outperformed competing models [10] on the Social Media Mining for Health (SMM4H) and Adverse Drug Event Corpus (CADEC) datasets developed by the Commonwealth Scientific and Industrial Research Organisation (CSIRO) [11,12]. Narayanan *et al.* [13] evaluated various biomedical contextual embeddings and models using the 2018 National NLP Clinical Challenges (n2c2) shared task Track 2 dataset on ADEs and medication extraction [14], demonstrating the great potential of the BERT architecture. In recent work on adverse event (AE) detection, Chopard *et al.* [15] confirmed the feasibility of automating the coding of AEs described in the narrative section of serious AE report forms. Furthermore, Silverman *et al.* [16] demonstrated that large language models (LLMs), such as University of California, San Francisco (UCSF)-BERT, achieved higher accuracy in detecting serious AEs from clinical notes compared with previous methods. A review [17] of machine learning and deep learning approaches for ADE extraction using benchmark datasets, including n2c2 [14], highlighted BERT’s superior performance in end-to-end tasks. A scoping review by Golder *et al.* [18] demonstrated the potential of NLP for identifying pediatric ADEs from clinical notes, while also highlighting challenges related to methodological heterogeneity, limited external validation, and inconsistent reporting. A narrative review by Zitu *et al.* [19] summarized the current state of LLMs, including BERT and the Generative Pretrained Transformer (GPT) models, in ADE detection, showing that LLM-based methods often outperform traditional approaches, although limitations such as performance variability and interpretability challenges remain.

Despite the good performance of BERT model and its variants (hereafter referred to as BERT Short-Formers, in contrast to Longformer [20]), one limitation is that long texts may be truncated due to token limits. To mitigate this limitation, Longformer [20], a BERT variant that can handle long text sequences, was developed. The key innovation of Longformer is its attention mechanism, which considers window-based local-context self-attention and global attention to achieve powerful contextual and sequential representations.

Our work began with exploring effective data processing methods for extracting information from clinical notes in EHRs for transformer-based models, ultimately aiming to develop an NLP system for detecting ADEs in clinical notes. To achieve this goal, we systematically investigated two data processing methods, called window-based and split-based approaches, to generate model inputs for fine-tuned BERT Short-Formers (BERT-base-uncased [5], a general-domain pretrained language model; BioMed-RoBERTa [21], a RoBERTa-based model further pre-trained on biomedical text; BioClinicalBERT [22], a BERT model further pre-trained on both biomedical literature and clinical notes; BioBERT [23], a BERT model further pre-trained on biomedical literature; PubMedBERT [24], a BERT model pre-trained on PubMed abstracts and full-text biomedical articles; and SpanBERT [9], a BERT model pre-trained with span-based masking objectives), as well as Clinical-Longformer [25,26], a Longformer-based model further pre-trained on clinical text, using the n2c2 dataset. We then applied the optimal processing and modeling methods to a dataset extracted from Vanderbilt University Medical Center (VUMC) EHRs to further assess generalizability. The two datasets contain markedly different types of clinical notes: the n2c2 dataset comprises fewer but longer notes, with a higher number of ADEs derived from hundreds of drug mentions, whereas the VUMC dataset includes more notes of shorter length, featuring more homogeneous ADEs focused on two drugs of interest. By applying these differing approaches to two distinct datasets, we aim to provide guidance for identifying ADEs from clinical notes using transformer-based models.

## Materials and methods

### Data sources

#### The n2c2 dataset.

The n2c2 dataset was derived from the 2018 n2c2 shared task Track 2 [14,27–31], sourced from the MIMIC-III (Medical Information Mart for Intensive Care III) database [32]. It includes 505 discharge summaries from a predominantly adult population, annotated with drug names, ADEs, and their relationships, split into 303 training and 202 test notes. In the training set, 78.2% of notes contained ADEs, with 321 of approximately 2,220 (14.5%) unique drugs (not counting misspellings or inconsistent naming), and 864 of 16,223 (5.3%) drug mentions associated with at least one ADE. The test set showed a similar distribution, with 76.7% of notes containing ADEs, 241 of approximately 1,676 (14.4%) unique drugs, and 606 of 10,575 (5.7%) drug mentions associated with at least one ADE.

#### The VUMC dataset.

The VUMC dataset was extracted from the Synthetic Derivative, de-identified EHRs at VUMC and was previously used in another study [33], which included a cohort of pediatric outpatients with ADEs related to two drugs of interest (citalopram/Celexa, escitalopram/Lexapro). As gold standard labels were not available, we performed manual annotation as described below. The final cohort included 112 patients with a total of 1,541 notes that mentioned at least one drug of interest, split into: 1,109 notes in the training set, with 12.2% of notes containing ADEs, and 284 of 1,910 (14.9%) drug mentions being ADE-associated, and 432 notes in the test set, with 8.3% of notes containing ADEs, and 59 of 757 (7.8%) drug mentions being ADE-associated.

### Annotation of the VUMC dataset

To create gold standard labels for the VUMC dataset, we used the brat rapid annotation tool (BRAT) [34] to manually annotate AEs associated with drugs in clinical notes. Before labelling, we formulated an initial set of annotation labels and corresponding guidelines. These guidelines and labels were updated through an iterative process where a few notes were annotated, discussed, and reannotated within a small group of researchers.

Specifically, we started with detailed labels to capture nuances of clinical context: AEpositive, AEnegative, AEpositiveNoJustification, AEconditional, and NoResponse. Drug names were labeled as AEpositive if they were associated with an ADE, AEnegative if they were not associated with an ADE, AEpositiveNoJustification if they were associated with a change in dose due to an ADE but without explicit justification, AEconditional if they were associated with a possible change in dose due to an ADE under certain conditions (e.g., if symptoms such as weight loss worsened while on the medication), and NoResponse if they were associated with a change in dose due to non-response to drug (i.e., the drug is not working), but not due to an ADE. After completing the annotation, we randomly selected a subset of 300 notes to validate the annotation by two independent annotators and evaluated agreement, yielding a Cohen’s Kappa statistic of 0.820. Excluding each label category individually did not change Kappa meaningfully, indicating consistent agreement across all categories. Discrepancies between annotators were resolved through group discussion to establish the final gold standard.

For the ADE classification task, the gold standard label “ADE positive” was constructed by combining AEpositive, AEpositiveNoJustification, and AEconditional, while “ADE negative” combined AEnegative and NoResponse. AEconditional was categorized as positive because it indicates a conditionally present or implied ADE, thereby capturing potential signals. In contrast, NoResponse was categorized as negative because it reflects a lack of drug efficacy rather than the occurrence of an ADE, which was confirmed during annotation.

### Data processing

Pre-trained transformer models can only take a limited length of free text, typically up to 512 tokens, whereas the Longformer can take up to 4,096 tokens. As clinical notes are typically long and unstructured, requiring preprocessing prior to modeling. We investigated several processing methods, ultimately focusing on the following two.

#### Window-based approach.

When drug names of interest are already annotated or can be easily located, this approach can be useful to effectively capture the contextual details surrounding drug names. It extracts a specific number of words before and after the drug name, called *window size*, by leveraging whitespace-based tokenization; for example, 20 words before and after *vancomycin*, a drug of interest in the n2c2 dataset. The window sizes we investigated include 10, 15, 20, 50, and 100 words. If the number of words before or after the drug name was insufficient, we extracted words up to the beginning or end of the note.

#### Split-based approach.

We explored a simpler alternative for settings where drug names are not yet annotated, as identifying them requires substantial effort. This approach exclusively partitions clinical notes into predefined *chunk sizes,* such as 2, 4, 10, and 20 chunks. We first used the Natural Language Toolkit (NLTK) tokenizer [35] to tokenize the text into sentences, and then divided the sentences into predefined chunk size. If the token length of a particular chunk exceeded a predefined token limit (e.g., 4,096 for Longformer), we further divided it into two smaller chunks at the word level. This additional step ensured that the chunks remained within the desired token limit, preventing truncation of context. Thereafter, no further splitting was required as most chunks were within the token limit. However, a small number of chunks still exceeded the token limit due to missing punctuation, especially at the beginning or end of some clinical notes.

**Fig 1:**
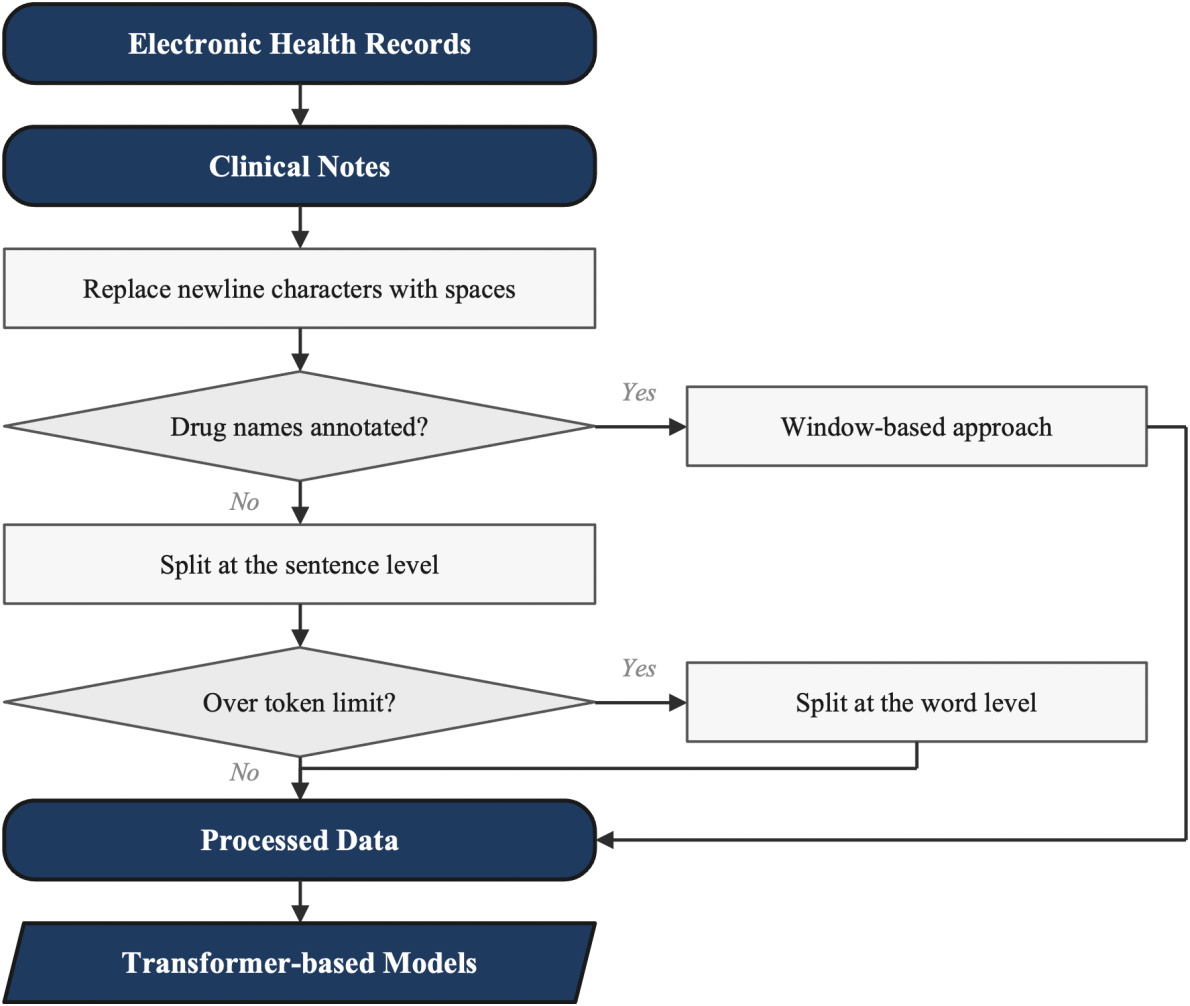
Schematic diagram of data processing steps. Window-based approach: extracts a predefined number of words before and after annotated drug names. Split at the sentence level: divides clinical notes into predefined chunk sizes at sentence boundaries. Split at the word level: subdivides any chunk exceeding the token limit into two smaller chunks at word boundaries.

For both approaches, newline characters were replaced with spaces to facilitate processing. A schematic diagram illustrating the data processing steps is shown in Fig 1. Examples of each strategy are provided in the Supporting Information.

### Computational environment

All experiments were conducted using the Hugging Face framework [36] on NVIDIA A100 40GB GPUs with Python 3.12.4 and Transformers 4.57.3.

### Model specifications and fine-tuning

BERT Short-Formers and Clinical-Longformer were fine-tuned using a per-device batch size of 16 with gradient accumulation of 2. Mixed precision training (FP16) and gradient checkpointing were employed to reduce memory usage and improve training efficiency. BERT Short-Formers were trained for 3 epochs with early stopping (patience = 2, minimum delta = 0.001), while all other hyperparameters were retained at their default settings. Clinical-Longformer used dataset-specific attention windows based on text length, with the n2c2 dataset trained using the default attention window of 512 and the VUMC dataset using a window of 128 and a warmup ratio of 0.1. Training for Clinical-Longformer was conducted for 10 epochs, with all other hyperparameters consistent with those used for BERT Short-Formers. A fixed random seed was used across all experiments to ensure reproducibility. To address class imbalance, we explored mitigation strategies such as class weighting and focal loss. However, these approaches did not significantly improve performance and were therefore not implemented.

### Model evaluation

We used the original n2c2 train and test splits to allow direct comparison with prior studies. 5-fold cross-validation was performed on the training set for model development, using 4 folds for training and 1 for validation, with per-fold threshold tuning. The hold-out test set was reserved exclusively for final evaluation and was not used during training or tuning. The VUMC dataset followed the same protocol.

Given the extreme class imbalance in ADE cases, area under the precision-recall curve (AUPRC) and macro F1 score were selected as the primary metrics, supplemented by micro F1, macro precision, and macro recall. Means and standard deviations (SDs) for all metrics on the test set are reported.

## Results

Table 1 presents the results of the fine-tuned BERT-base-uncased model on the n2c2 dataset using varying window sizes. Window sizes of 10–20 words performed well, with a 15-word window achieving the best results (mean ± SD: AUPRC 0.670 ± 0.006, micro F1 0.963 ± 0.001, macro F1 0.804 ± 0.005). This suggests that a 15-word window around the drug name provides sufficient context about ADEs associated with the drug. Our exploratory analysis regarding the distance between drug names and ADEs supported that a 15-word window covered approximately 80% of ADEs. Wider window sizes yielded poorer performance, likely due to the inclusion of longer and more complex contextual information (e.g., medical history and prescriptions), which may introduce noise. Although broader windows capture more context, they do not necessarily improve model performance. Conversely, excessively small windows may exclude ADEs. The SDs of the performance metrics were small across window sizes, indicating stable results. Given that our goal is to identify as many positive cases as possible, a moderate window size offers a better balance.

**Table 1:** Effects of window size on BERT-base-uncased model performance using the n2c2 dataset.

Window Size	AUPRC	Micro F1	Macro Precision	Macro Recall	Macro F1
10-word	0.646 (0.015)	0.961 (0.001)	0.843 (0.007)	0.759 (0.019)	0.794 (0.011)
15-word	**0.670 (0.006)**	**0.963 (0.001)**	**0.851 (0.006)**	**0.770 (0.009)**	**0.804 (0.005)**
20-word	0.658 (0.023)	0.960 (0.002)	0.829 (0.016)	0.770 (0.016)	0.796 (0.009)
50-word	0.477 (0.071)	0.949 (0.003)	0.775 (0.020)	0.667 (0.042)	0.703 (0.042)
100-word	0.306 (0.119)	0.942 (0.002)	0.674 (0.102)	0.593 (0.047)	0.617 (0.066)

Values are reported as mean (standard deviation) from 5-fold cross-validation.

To leverage domain-specific knowledge, we fine-tuned several BERT Short-Formers pre-trained on biomedical corpora using the optimal 15-word window. Performance was evaluated on the n2c2 dataset (Table 2). BioClinicalBERT, BioBERT, PubMedBERT, and Clinical-Longformer demonstrated strong performance. Among them, PubMedBERT achieved the highest AUPRC (0.698 ± 0.018), followed by Clinical-Longformer (0.689 ± 0.058). These findings suggest that biomedical pre-training enhances a model’s ability to capture domain-specific information and improves classification performance. Therefore, in the subsequent evaluation, we selected PubMedBERT as the best-performing BERT Short-Former, BERT-base-uncased as the baseline, and Clinical-Longformer as the representative Longformer model.

**Table 2:** Model comparison with window size of 15 words using the n2c2 dataset.

Model	AUPRC	Micro F1	Macro Precision	Macro Recall	Macro F1
BERT-base-uncased	0.670 (0.006)	0.963 (0.001)	0.851 (0.006)	0.770 (0.009)	0.804 (0.005)
BioMed-RoBERTa	0.663 (0.055)	0.961 (0.003)	0.843 (0.018)	0.763 (0.028)	0.796 (0.020)
BioClinicalBERT	0.684 (0.029)	0.965 (0.001)	0.857 (0.007)	**0.794 (0.011)**	**0.822 (0.007)**
BioBERT	0.681 (0.008)	**0.965 (0.000)**	**0.858 (0.003)**	0.790 (0.007)	0.820 (0.004)
PubMedBERT	**0.698 (0.018)**	0.963 (0.001)	0.854 (0.008)	0.779 (0.012)	0.811 (0.007)
SpanBERT	0.381 (0.221)	0.947 (0.006)	0.659 (0.156)	0.637 (0.119)	0.642 (0.130)
Clinical-Longformer	0.689 (0.058)	0.963 (0.004)	0.849 (0.020)	0.787 (0.028)	0.814 (0.024)

Values are reported as mean (standard deviation) from 5-fold cross-validation.

The results of text partitioning for the two selected BERT Short-Formers and Clinical-Longformer on the n2c2 dataset are presented in Table 3. The number of chunks (i.e., chunk size) substantially affects model performance by determining the extent of text truncation imposed by token limits. Clinical-Longformer performed consistently well across most configurations: the 4-chunk setting achieved the best AUPRC (0.840 ± 0.056), the 20-chunk setting obtained the best micro F1 (0.939 ± 0.006), and the 10-chunk setting attained the best macro F1 (0.852 ± 0.007). We also compared two BERT Short-Formers and Clinical-Longformer using 10- and 20-chunk configurations on the n2c2 dataset. These chunk sizes were selected because most chunks remained within the token limit of BERT Short-Formers, given that the 95th percentile word count in the n2c2 training set was approximately 1,368. Results for 2- and 4-chunk settings were not reported for the BERT Short-Formers as most segments would exceed the token limits. With 10 chunks, the models achieved AUPRCs of 0.635 ± 0.060 (BERT-base-uncased), 0.745 ± 0.028 (PubMedBERT), and 0.819 ± 0.017 (Clinical-Longformer). With 20 chunks, the corresponding AUPRCs were 0.624 ± 0.023 (BERT-base-uncased), 0.733 ± 0.023 (PubMedBERT), and 0.757 ± 0.043 (Clinical-Longformer), respectively. The SDs for Clinical-Longformer with the 2- and 4-chunk settings were slightly higher than those of the other configurations, although the differences were modest. Overall, these findings highlighted the importance of selecting an appropriate partitioning strategy to preserve context integrity and optimize model performance.

**Table 3:** Effects of split chunk size on model performance using the n2c2 dataset.

Model	Chunk Size	AUPRC	Micro F1	Macro Precision	Macro Recall	Macro F1	Proportion over Token Limit
BERT-base-uncased	2 chunks	–	–	–	–	–	–
BERT-base-uncased	4 chunks	–	–	–	–	–	–
BERT-base-uncased	10 chunks	0.635 (0.060)	0.866 (0.017)	0.763 (0.032)	0.754 (0.019)	0.756 (0.020)	7.38%
BERT-base-uncased	20 chunks	0.624 (0.023)	0.919 (0.010)	0.799 (0.034)	0.745 (0.033)	0.764 (0.016)	2.65%
PubMedBERT	2 chunks	–	–	–	–	–	–
PubMedBERT	4 chunks	–	–	–	–	–	–
PubMedBERT	10 chunks	0.745 (0.028)	0.905 (0.004)	0.845 (0.016)	0.808 (0.028)	0.823 (0.011)	6.10%
PubMedBERT	20 chunks	0.733 (0.023)	0.938 (0.003)	0.848 (0.013)	0.803 (0.017)	0.823 (0.009)	2.19%
Clinical-Longformer	2 chunks	0.785 (0.062)	0.682 (0.042)	0.704 (0.028)	0.659 (0.050)	0.648 (0.062)	0.24%
Clinical-Longformer	4 chunks	**0.840 (0.056)**	0.811 (0.044)	0.802 (0.047)	0.802 (0.047)	0.800 (0.046)	0.00%
Clinical-Longformer	10 chunks	0.819 (0.017)	0.908 (0.002)	**0.855 (0.009)**	**0.851 (0.020)**	**0.852 (0.007)**	0.00%
Clinical-Longformer	20 chunks	0.757 (0.043)	**0.939 (0.006)**	0.850 (0.020)	0.838 (0.036)	0.842 (0.020)	0.00%

Values are reported as mean (standard deviation) from 5-fold cross-validation.

We further conducted statistical testing using the Wilcoxon signed-rank test on paired results from 5-fold cross-validation to compare Clinical-Longformer (4-chunk setting) and PubMedBERT (10-chunk setting), which achieved the highest AUPRCs. The resulting p-values were 0.0625 for AUPRC, 0.0625 for micro F1, and 0.4375 for macro F1, indicating no statistically significant differences at the level of 0.05. However, the small number of folds (i.e., limited sample size) constrained the statistical power of these tests. Notably, for a paired sample size of 5 with no ties or zero differences, the minimum achievable p-value under a two-sided exact Wilcoxon signed-rank test is 0.0625. Thus, even if all paired AUPRC and macro F1 values for Clinical-Longformer exceeded those of PubMedBERT, statistical significance could not be reached. To further examine this limitation, we conducted additional analysis using 10-fold cross-validation. This yielded p-values of 0.0020 for AUPRC, 0.0020 for micro F1, and 0.1055 for macro F1. In this setting, 0.0020 represents the minimum possible p-value for a paired sample size of 10, consistent with all AUPRC values for Clinical-Longformer exceeding those of PubMedBERT. These findings suggested a potential performance difference between the models. However, we did not adopt 10-fold cross validation for all analyses, as it resulted in smaller validation sets, which may be suboptimal given the sample sizes. Therefore, we retained 5-fold cross-validation throughout and reported mean and SD values, without additional formal statistical testing due to the limited statistical power.

Table 4 shows the performance of the two selected BERT Short-Formers and Clinical-Longformer for classifying ADEs in the VUMC dataset. As clinical notes in the VUMC dataset are relatively short, we evaluated chunk sizes of 1 (i.e., entire note, so no split), 2, 4 and 6 chunks. The results of 1-chunk with BERT Short-Formers are not reported due to convergence issues caused by token limit exceedance. Although most VUMC notes fall within the token limit of Longformer, the 2-chunk, 4-chunk, and 6-chunk outperformed 1-chunk. This suggests that moderately partitioned text may provide more focused contextual information for ADE detection. Similar trends were obtained with BERT-base-uncased and PubMedBERT. Furthermore, Clinical-Longformer substantially outperformed BERT Short-Formers, likely due to its ability to process much longer token sequences and preserve more complete contextual information. However, increasing the number of chunks to 6 did not consistently improve performance across all models. Further partitioning may increase the chance that the ADE and the corresponding drug name are placed in different chunks, fragmenting contextual information. Excessive partitioning can therefore reduce performance by limiting a model’s ability to capture comprehensive context and introducing additional complexity.

**Table 4:** Effects of split chunk size on model performance using the VUMC dataset.

Model	Chunk Size	AUPRC	Micro F1	Macro Precision	Macro Recall	Macro F1	Proportion over Token Limit
BERT-base-uncased	1 chunk	–	–	–	–	–	–
BERT-base-uncased	2 chunks	0.341 (0.041)	0.932 (0.016)	0.670 (0.043)	0.663 (0.025)	0.661 (0.025)	20.60%
BERT-base-uncased	4 chunks	0.380 (0.055)	0.958 (0.007)	0.722 (0.068)	0.687 (0.041)	0.690 (0.014)	7.36%
BERT-base-uncased	6 chunks	0.472 (0.054)	**0.966 (0.004)**	0.709 (0.032)	0.752 (0.054)	0.720 (0.017)	4.76%
PubMedBERT	1 chunk	–	–	–	–	–	–
PubMedBERT	2 chunks	0.311 (0.049)	0.936 (0.018)	0.703 (0.081)	0.622 (0.030)	0.636 (0.019)	19.20%
PubMedBERT	4 chunks	0.390 (0.144)	0.954 (0.013)	0.695 (0.078)	0.679 (0.050)	0.682 (0.054)	6.60%
PubMedBERT	6 chunks	0.342 (0.139)	0.963 (0.005)	0.627 (0.072)	0.668 (0.091)	0.644 (0.078)	4.29%
Clinical-Longformer	1 chunk	0.291 (0.070)	0.840 (0.040)	0.611 (0.042)	0.629 (0.040)	0.611 (0.036)	0.00%
Clinical-Longformer	2 chunks	0.373 (0.097)	0.907 (0.010)	0.668 (0.019)	0.709 (0.068)	0.679 (0.030)	0.00%
Clinical-Longformer	4 chunks	0.532 (0.118)	0.954 (0.014)	**0.736 (0.050)**	0.808 (0.014)	**0.762 (0.035)**	0.00%
Clinical-Longformer	6 chunks	**0.536 (0.147)**	0.963 (0.014)	0.725 (0.062)	**0.822 (0.035)**	0.759 (0.052)	0.00%

Values are reported as mean (standard deviation) from 5-fold cross-validation.

## Discussion

We fine-tuned pre-trained transformer-based models for detecting ADEs from two distinct datasets. Models pre-trained on biomedical corpora demonstrated strong capability in ADE classification tasks by leveraging domain-specific knowledge. Among BERT Short-Formers, PubMedBERT achieved the best performance (AUPRC = 0.745, micro F1 = 0.963, macro F1 = 0.823 in the best-performing setting) on the n2c2 dataset. Clinical-Longformer generally outperformed BERT Short-Formers across most metrics on the n2c2 dataset (AUPRC = 0.840, micro F1 = 0.963, macro F1 = 0.852 in the best-performing setting) and the VUMC dataset (AUPRC = 0.536, micro F1 = 0.963, macro F1 = 0.762 in the best-performing setting), suggesting that the ability to model longer clinical context further enhances performance. To the best of our knowledge, this is the first study to fine-tune the Longformer model for ADE detection, demonstrating its applicability to clinical NLP tasks.

Because transformer models are constrained by token limits, we systematically evaluated window-based and split-based text processing strategies. The window-based approach performed optimally with a 15-word window, indicating that moderate contextual scope effectively balances information retention and noise reduction. The split-based approach was introduced as a practical alternative that does not require annotated drug names, as such annotation demands significant effort. We found that chunk size significantly affected performance, even when note length was within the token limit. Moderate partitioning improved results, whereas excessive partitioning reduced performance, likely due to fragmentation of contextual information. Small SDs across folds indicated stable model performance. Comparisons between architectures further highlighted important differences. BERT-base-uncased performed better with the window-based processing approach, likely due to its focused contextual learning around drug mentions. Clinical-Longformer benefited more from split-based processing, consistent with its design for longer sequences. These findings emphasize that optimal ADE detection depends on the joint selection of text partitioning and model architecture strategies.

There are a few prior studies on the n2c2 dataset focused on ADE-Drug relation using BERT-based models. Our models achieved similar or improved performance (micro F1 score of 0.96 on BERT-base-uncased, 0.97 on BioClinicalBERT and BioBERT) compared to previously reported results by Wei *et al.* (micro F1 score of 0.80 on BERT-large-uncased and 0.81 on MIMIC BERT) [5,6,37], and Mahendran *et al.* (micro F1 scores of 0.97 on BERT-base-uncased, BioClinicalBERT and BioBERT) [3]. In a recent work, Dai *et al.* [38] reported micro F1 scores (SDs) of 0.25, 0.52 (0.01), and 0.15 (0.22) on GPT-4, fine-tuned span-based models, and fine-tuned generative models, respectively, including generative artificial intelligence (AI) based approaches. Due to substantial class imbalance, we used AUPRC and macro metrics as the primary evaluation measures, as they better reflect performance on the minority class than micro F1 that is equivalent to accuracy in binary classification. Micro F1 scores were uniformly high and did not meaningfully differentiate model performance.

On institutional datasets, Silverman *et al.* [16] reported accuracy of 0.88–0.92 and macro F1 of 0.61–0.68 in drug-serious AE pair identification from annotated outpatient clinical notes at UCSF using Hierarchical UCSF BERT (H-UCSF-BERT). In comparison, our results on the VUMC dataset demonstrated improved performance with an accuracy of 0.97 using BERT-base-uncased, and a macro F1 of 0.76 using Clinical-Longformer. It is important to note that their notes focused on 8 non-steroid immunosuppressants, whereas our study using the VUMC dataset focused specifically on AEs associated with two medications, citalopram and escitalopram. Although we also evaluated our approaches on the n2c2 dataset, which contains a broader set of drug-ADE relations, direct comparison was not possible because Silverman *et al.* did not report H-UCSF-BERT results on the n2c2 dataset.

When using non-default attention windows with Clinical-Longformer, we occasionally observed training instability. In some runs, the model failed to converge during the early epochs despite adjustments to the warmup ratio and learning rate. In other cases, models converged but exhibited lower validation and test performance and greater variability across runs. These effects may be related to disrupted gradient flow when attention windows are too small, attention sink behavior where certain tokens absorb disproportionate attention, or task-specific threshold effects when local context becomes insufficient. Overall, this suggests that the default attention window is empirically tuned for stable training, and non-default configurations may require substantial additional tuning. Nevertheless, transformer-based models, particularly Clinical-Longformer, remain valuable for facilitating the extraction of meaningful signals from clinical notes, with their advantages outweighing potential challenges.

This study has several limitations. First, limited hyperparameter tuning of the transformer-based models may have constrained full exploration of their performance. Future work could investigate a broader range of training configurations. Second, this study utilized the n2c2 dataset and a single institution-specific dataset. These two datasets are inherently distinct, reflecting substantially different clinical contexts, documentation practices, medication coverage, and patient populations, and thus provide meaningful heterogeneity that supports the assessment of model generalizability across varied healthcare settings. Despite that, validation across additional datasets, institutions, and drug classes remains warranted, particularly for medications with rare or heterogeneous AE profiles. Institution-specific models such as UCSF-BERT, if made publicly available, represent promising avenues for future comparative studies, as they may offer further insight into model performance across diverse institutional note structures and patient populations, thereby strengthening the assessment of generalizability. Third, potential annotation bias may have introduced variability into the labeled data due to differences among annotators, training procedures, and human error. Finally, although we observed qualitative trade-offs between human annotation effort (i.e., the requirement for window-based vs. no requirement for split-based processing) and computational resources (e.g., runtime for BERT Short-Formers vs. Longformer), quantitative comparison was not feasible due to the lack of systematically recorded annotation time and computational costs.

## Conclusion

In this study, we presented transformer-based models for detecting ADEs as well as different data processing methods and their impact on model performance. Window-based processing approaches rely heavily on annotations, which is labor-intensive, but can build precise context when the distances between drug names and their associated ADEs in the dataset are relatively consistent. Split-based processing approaches can easily split long texts into smaller chunks that transformer models can handle and do not require labor-intensive annotation for drug-ADE relations. However, chunk size determines the proportion of chunks that exceed the token limit (Tables 3 and 4), thereby affecting the classification task, and identifying the optimal chunk size can be challenging. The Longformer model requires more extensive fine-tuning, including but not limited to memory, runtime and hyperparameter settings, while for BERT Short-Formers, token limit is the major challenge.

Although our results are promising, the limitations highlight directions for further research and improvement to enhance the applicability and generalizability of transformer-based NLP models for ADE detection in real-world healthcare systems.

For future work, we aim to extend our findings to additional datasets and a broader range of drugs, enabling more comprehensive validation across diverse clinical scenarios. To improve the extraction of ADE-related information from EHRs, we plan to incorporate additional elements such as symptoms, actions, and dose changes, which may enhance understanding of drug-ADE relations and support clinical decision-making. Moreover, data augmentation strategies such as paraphrasing, synthetic data generation, oversampling, along with advanced long-document modeling techniques, including hybrid approaches that combine window- and split-based approaches, retrieval-augmented methods, and dual-pass architectures, could be explored to more effectively capture contextual information for ADE tasks. Continued methodological development in this area may contribute to more robust NLP-based ADE detection systems and ultimately support improvements in patient safety and health outcomes.

## Supporting information

Table S1 Examples of text partitioning strategies using a synthetic clinical note. (DOCX)
