# Supplementary material for: Developing a natural language processing system using transformer-based models for adverse drug event detection in electronic health records
Source: PLoS One. 2026 Jul 1;21(7):e0350516. doi: 10.1371/journal.pone.0350516 (PMC13322497; doi:10.1371/journal.pone.0350516)
Supplement: Table S1 — (DOCX) [file pone.0350516.s001.docx]

**Supporting Information**

Table S1 presents examples of the three text partitioning strategies applied to a synthetic clinical note around the target drug ***metformin***.

**Table S1. Examples of Text Partitioning Strategies.**

**Input Note**

| A 62-year-old woman with a history of hypertension and obesity presented to the outpatient clinic for a routine diabetes management visit. She reported increased fatigue and frequent urination over the preceding four weeks. Review of systems was otherwise unremarkable, with no chest pain, dyspnea, or lower extremity edema. Her blood pressure was 138/86 mmHg and body mass index was 31.4 kg/m². Her primary care provider had initiated ***metformin*** six months prior for newly diagnosed type 2 diabetes mellitus. Adherence had been inconsistent due to gastrointestinal side effects, including nausea and bloating. Hemoglobin A1c was 8.9%, indicating suboptimal glycemic control. The treatment plan was revised to include a lower starting dose and dietary counseling. Follow-up laboratory testing and a repeat clinic visit were scheduled for eight weeks. |
| --- |

|  | **Window-Based** | **Sentence-Level Split** | **Word-Level Split** |
| --- | --- | --- | --- |
| **How it works** | Extracts a fixed window of words immediately surrounding the target drug name. | Divides the full note into predefined chunks aligned to sentence boundaries. | Divides the full note into predefined chunks aligned to word boundaries. |
| **Output Chunk 1** | 138/86 mmHg and body mass index was 31.4 kg/m². Her primary care provider had initiated  ***metformin*** six months prior for newly diagnosed type 2 diabetes mellitus. Adherence had been inconsistent due | A 62-year-old woman with a history of hypertension and obesity presented to the outpatient clinic for a routine diabetes management visit. She reported increased fatigue and frequent urination over the preceding four weeks. Review of systems was otherwise unremarkable, with no chest pain, dyspnea, or lower extremity edema. Her blood pressure was 138/86 mmHg and body mass index was 31.4 kg/m². Her primary care provider had initiated ***metformin*** six months prior for newly diagnosed type 2 diabetes mellitus. | A 62-year-old woman with a history of hypertension and obesity presented to the outpatient clinic for a routine diabetes management visit. She reported increased fatigue and frequent urination over the preceding four weeks. Review of systems was otherwise unremarkable, with no chest pain, dyspnea, or lower extremity edema. Her blood pressure was 138/86 mmHg and body mass index was 31.4 kg/m². Her primary care provider had initiated ***metformin*** six months prior for newly diagnosed type 2 diabetes mellitus. Adherence had been |
| **Output Chunk 2** | — | Adherence had been inconsistent due to gastrointestinal side effects, including nausea and bloating. Hemoglobin A1c was 8.9%, indicating suboptimal glycemic control. The treatment plan was revised to include a lower  starting dose and dietary counseling. Follow-up laboratory testing and a repeat clinic visit were scheduled for eight weeks. | inconsistent due to gastrointestinal side effects, including nausea and bloating. Hemoglobin A1c was 8.9%, indicating suboptimal glycemic control. The treatment plan was revised to include a lower starting dose and dietary counseling. Follow-up laboratory testing and a repeat clinic visit were scheduled for eight weeks. |
