# Supplementary material for: Developing a natural language processing system using transformer-based models for adverse drug event detection in electronic health records
Source: PLoS One. 2026 Jul 1;21(7):e0350516. doi: 10.1371/journal.pone.0350516 (PMC13322497; doi:10.1371/journal.pone.0350516)
Supplement: Attachment — Submitted filename: AEproject_Response_to_Reviewers_0411.docx [file pone.0350516.s003.docx]

**Point-by-Point Response to Reviewers’ Comments**

April 12, 2026

Manuscript ID: PONE-D-25-22628

Title: Developing a natural language processing system using transformer-based models for adverse drug event detection in electronic health records

We thank the editor and the reviewers for the insightful comments and suggestions, which has helped us significantly improve our manuscript. We have carefully considered all comments and have addressed the majority of the requested revisions. Where certain suggestions could not be fully implemented, we have provided detailed explanations and clarifications in our responses. Please see the itemized responses below.

**Editor Comments:**

*The comments of the reviewers ask for some important modifications and additional work to improve your manuscript, such as state-of-the-art additional comparison, deeper analysis of the results, as well as better image quality. So, I encourage the authors to consider all these suggestions and resubmit your work.*

**Response:** We thank you for the comment. As suggested, we have carefully considered all comments and substantially strengthened the manuscript based on the suggestions.

We expanded the discussion to include comparison with additional state-of-the-art models, including generative artificial intelligence (AI) approaches and institution-specific models, to better contextualize our findings. We also reran all analyses using 5-fold cross-validation and reported more comprehensive performance metrics. To improve clarity and readability, we have revised Fig 1 by splitting it into two parts. While the diagram has been retained, we have moved the examples of text partitioning strategies to the Supporting Information to reduce visual clutter and enhance interpretability. In addition, we have regenerated the figure at higher resolution for the revised manuscript.

To explore the possibility of including University of California, San Francisco Bidirectional Encoder Representations from Transformer (UCSF-BERT) in our comparison, we contacted Dr. Vivek Rudrapatna, the principal investigator, to request access. We were informed that the model is not publicly available and requires execution of a Data Use Agreement (DUA) to obtain access. While we appreciate the opportunity to request access, the DUA includes legal provisions that extend obligations beyond the project period (e.g., Section 5: “All of Recipient’s obligations herein shall survive the expiration or termination of this Agreement indefinitely.”). Given these requirements, we were unable to proceed with the agreement. We therefore focused our comparisons on publicly available BERT-based models, which facilitate reproducibility and broader use by the research community. We believe this provides a fair and practical comparison across the two datasets, which differ substantially in clinical contexts, documentation practices, medication coverage, and patient populations. To acknowledge the reviewer’s point, we have expanded the discussion about UCSF-BERT’s work in the revised manuscript and noted UCSF-BERT and other institution-specific models as potential directions for future comparative studies when access becomes available.

**Reviewer #1:**

*Did you consider hybrid approaches that might combine advantages of both window-based and split-based processing methods?*

**Response:** We thank you for the comment. In this study, our primary objective is to evaluate window-based and split-based strategies independently to clearly understand their respective effects. We agree that hybrid approach is an interesting direction; however, it is beyond the scope of the current work. We have now acknowledged hybrid approaches as a promising avenue for future research in the Conclusion section of the revised manuscript, as follows:

“Moreover, data augmentation strategies such as paraphrasing, synthetic generation, oversampling, along with advanced long-document modeling techniques, including hybrid approaches that combine window- and split-based approaches, retrieval-augmented methods, and dual-pass architectures, could be explored to more effectively capture contextual information for ADE tasks.”

*Could you elaborate on the specific patterns of instability observed with Clinical-Longformer's non-default attention windows (e.g., types of performance drops or convergence failures)?*

**Response:** We have now elaborated on the specific patterns of instability in the Discussion section of the revised manuscript, as follows:

“When using non-default attention windows with Clinical-Longformer, we occasionally observed training instability. In some runs, the model failed to converge during the early epochs despite adjustments to the warmup ratio and learning rate. In other cases, models converged but exhibited lower validation and test performance and greater variability across runs. These effects may be related to disrupted gradient flow when attention windows are too small, attention sink behavior where certain tokens absorb disproportionate attention, or task-specific threshold effects when local context becomes insufficient. Overall, this suggests that the default attention window is empirically tuned for stable training, and non-default configurations may require substantial additional tuning.”

*Were there particular label categories in the annotation process that showed lower agreement than the overall Kappa of 0.820, and how were these resolved?*

**Response:** To assess whether any specific label categories exhibited lower agreement, we recalculated Kappa after excluding each category individually. None of these exclusions led to a meaningful change in the overall Kappa of 0.820, indicating that inter-annotator agreement was generally consistent across all categories. We have now included this clarification in the Materials and Methods section of the revised manuscript:

“After completing the annotation, we randomly selected a subset of 300 notes to validate the annotation by two independent annotators and evaluated agreement, yielding a Cohen’s Kappa statistic of 0.820. Excluding each label category individually did not change Kappa meaningfully, indicating consistent agreement across all categories. Discrepancies between the annotators were discussed as a group and a final decision was made about what the gold standard should be.”

*Did you perform statistical tests to determine if the performance differences between models (e.g., Clinical-Longformer's 0.868 vs PubMedBERT's 0.832 F-score) were significant?*

**Response:** Thank you for the comment. To assess whether the observed performance differences between models were statistically significant, we conducted a Wilcoxon signed-rank test using the paired evaluation metrics obtained from the 5-fold cross-validation. This non-parametric test does not assume normality and is commonly used for comparing the paired differences. The analysis showed that the performance difference between Clinical-Longformer (4-chunk setting) and PubMedBERT (10-chunk setting), which achieved the highest area under the precision-recall curves (AUPRCs), was not statistically significant, with p-values of 0.0625 for AUPRC, 0.0625 for micro F1, and 0.4375 for macro F1. We note that the limited number of cross-validation folds constrains the statistical power of these tests in general (i.e., p-values of 0.0625 is the minimum possible p-value for a paired sample size of 5); therefore, the results should be interpreted with this limitation in mind.

We further performed 10-fold cross-validation on both models and conducted the tests, yielding p-values of 0.0020 for AUPRC, 0.0020 for micro F1, and 0.1055 for macro F1, which suggested a potential difference in performance. However, we did not adopt 10-fold cross validation for all analyses, as it resulted in smaller validation sets, which may be suboptimal given the sample size. Therefore, we retained 5-fold cross-validation throughout and reported mean and standard derivation values, without additional formal statistical testing due to the limited statistical power. We have added this information to the Results section of the revised manuscript:

“We further conducted statistical testing using the Wilcoxon signed-rank test on paired results from 5-fold cross-validation to compare Clinical-Longformer (4-chunk setting) and PubMedBERT (10-chunk setting), which achieved the highest AUPRCs. The resulting p-values were 0.0625 for AUPRC, 0.0625 for micro F1, and 0.4375 for macro F1, indicating no statistically significant differences at the level of 0.05. However, the small number of folds (i.e., limited sample size) constrained the statistical power of these tests. Notably, for a paired sample size of 5 with no ties or zero differences, the minimum achievable p-value under a two-sided exact Wilcoxon signed-rank test is 0.0625. Thus, even if all paired AUPRC and macro F1 values for Clinical-Longformer exceeded those of PubMedBERT, statistical significance could not be reached. To further examine this limitation, we conducted additional analysis using 10-fold cross-validation. This yielded p-values of 0.0020 for AUPRC, 0.0020 for micro F1, and 0.1055 for macro F1. In this setting, 0.0020 represents the minimum possible p-value for a paired sample size of 10, consistent with all AUPRC values for Clinical-Longformer exceeding those of PubMedBERT. These findings suggested a potential performance difference between the models. However, we did not adopt 10-fold cross validation for all analyses, as it resulted in smaller validation sets, which may be suboptimal given the sample sizes. Therefore, we retained 5-fold cross-validation throughout and reported mean and SD values, without additional formal statistical testing due to the limited statistical power.”

*What specific measures were taken to ensure the evaluation wasn't skewed by the extreme class imbalance in the VUMC dataset (8.33-12.17% ADE-positive)?*

**Response:** To address the extreme class imbalance, we reported macro-averaged precision, recall, and F1 score, which balanced performance across classes. In this revision, we also included AUPRC and micro F1 to provide a more comprehensive evaluation. In addition, we used 5-fold cross-validation with per-fold threshold tuning to optimize performance for the minority class. We explored other imbalance mitigation strategies, including class weights and focal loss; however, preliminary experiments showed that these approaches did not noticeably improve performance and introduced additional variability. Therefore, threshold tuning combined with appropriate evaluation metrics was used in the final analysis. We have added this to the Materials and Methods section of the revised manuscript:

“To address class imbalance, we explored mitigation strategies such as class weighting and focal loss. However, these approaches did not significantly improve performance and were therefore not implemented.

…

5-fold cross-validation was performed on the training set for model development, using 4 folds for training and 1 for validation, with per-fold threshold tuning.

…

Given the extreme class imbalance in ADE cases, the area under the precision-recall curve (AUPRC) and macro F1 score were selected as the primary metrics, supplemented with micro F1, macro precision, and macro recall. Means and standard deviations (SDs) for all metrics on the test set are reported.”

*What range of variance was observed in performance metrics across the multiple trials with different seeds?*

**Response:** We thank the reviewer for this comment. To better quantify variability in model performance, we re-performed the analyses using 5-fold cross-validation and reported the mean and standard deviation for each evaluation metric. Across the five folds, we observed low variability, with a median standard deviation of 0.020 and an interquartile range (IQR) of 0.038 across different metrics and models, indicating consistent performance. The mean and standard deviation values for all evaluation metrics, calculated from the 5-fold cross-validation, are now reported in the revised manuscript. We also note that the results were similarly stable in our earlier analyses conducted using different random seeds, although these results are no longer reported in the revised manuscript.

*Could you provide more details about the VUMC data request process, including approval timelines and required documentation?*

**Response:** We made inquiry about this process to the team overseeing the use of Synthetic Derivative (SD) we used, which is a de-identified version of EHR data specifically developed for research. We were informed that all users should sign a Vanderbilt approved Data Use Agreement (DUA), which stipulates the user's responsibilities for maintaining the security of the data, prohibits any reidentification efforts, and mandates the reporting of residual identifiers that are found in the data. Researchers interested in accessing this dataset should contact the corresponding author, Leena Choi at leena.choi@vumc.org. We have added this information in the Data Availability and Code section of the revised manuscript:

“The VUMC dataset used in this study is not publicly available. Access requires a Vanderbilt-approved DUA, which outlines responsibilities for data security, prohibits reidentification, and mandates reporting of any residual identifiers. The data were collected under a waiver of consent, and access to the VUMC Synthetic Derivative, from which the data were extracted, requires Institutional Review Board (IRB) approval and a signed DUA for all investigators. Researchers interested in accessing this dataset should contact the corresponding author, Leena Choi at leena.choi@vumc.org.”

*Have you considered creating and sharing a synthetic version of the VUMC dataset that preserves clinical characteristics but removes PHI?*

**Response:** We appreciate the reviewer’s comment and agree that it would be good to share even a synthetic version of the dataset. We requested sharing a synthetic version of the data to the Vanderbilt University Medical Center (VUMC) team who developed and maintained the SD, de-identified EHRs for research. They responded that we are not allowed to share any version of this dataset due to the following reasons. The notes are already as de-identified as the team can make them using automatic processes. The only room for improvement is manual review and redaction, which is not feasible at scale and would not change the DUA requirement as mentioned above. The reason these records cannot be shared without a DUA is because they were collected under a waiver of consent and because the Institutional Review Board (IRB) approval for the SD requires a signed DUA from investigators who will access the data. The team provided us some language copied from the stamped IRB application below:

In the abstract section:

“Investigator access to this application, and future applications, requires IRB protocol review as well as a signed data use agreement that provides for protection of the data and prohibits reidentification.”

In a section outlining our responsibilities for protecting the data:

“Ensuring that all records released to researchers are protected by a data use agreement that stipulates how data can and cannot be used and expected safeguards.”

*Did you use the original n2c2 train/test splits from the shared task or create new splits?*

**Response:** We used the original n2c2 train and test splits to allow direct comparison with prior studies. 5-fold cross-validation was performed on the training set for model development, using 4 folds for training and 1 for validation. The hold-out test set was reserved exclusively for final evaluation and was not used during training or tuning. We have clarified this in the Materials and Methods section of the revised manuscript.

“We used the original n2c2 train and test splits to allow direct comparison with prior studies. 5-fold cross-validation was performed on the training set for model development, using 4 folds for training and 1 for validation, with per-fold threshold tuning. The hold-out test set was reserved exclusively for final evaluation and was not used during training or tuning. The VUMC dataset followed the same protocol.”

*Would consolidating model configuration details into a dedicated "Model Specifications" subsection improve readability?*

**Response:** Thank the reviewer for this helpful comment. We agree that a dedicated "Model Specifications" subsection improves readability. We have implemented this change in the Materials and Methods section of the revised manuscript, consolidating all relevant settings.

“**Computational Environment**

All experiments were conducted using the Hugging Face framework(36) on NVIDIA A100 40GB GPUs with Python 3.12.4 and the Transformers 4.57.3.

**Model Specifications and Fine-Tuning**

BERT Short-Formers and Clinical-Longformer were fine-tuned using a per-device batch size of 16 with gradient accumulation of 2. Mixed precision training (fp16) and gradient checkpointing were employed to reduce memory usage and improve training efficiency. BERT Short-Formers were trained for 3 epochs with early stopping (patience = 2, minimum delta = 0.001) and default hyperparameters. Clinical-Longformer used dataset-specific attention windows based on text length, with n2c2 trained using default attention window of 512 and the VUMC dataset using a window size of 128 and a warmup ratio of 0.1. Training for Clinical-Longformer was conducted for 10 epochs, with all other hyperparameters consistent with BERT Short-Formers. A fixed random seed was used across all experiments to ensure reproducibility. To address class imbalance, we explored mitigation strategies such as class weighting and focal loss. However, these approaches did not significantly improve performance and were therefore not implemented.”

*Could you add specific metrics (e.g., annotation time, compute hours) to quantify the "allocation trade-offs of human and computational power" mentioned in the abstract?*

**Response:** We agree that reporting quantitative measures such as annotation time and computation hours would further strengthen the assessment of human and computational trade-offs. However, these metrics were not systematically tracked during the study. Therefore, we are unable to retrospectively provide reliable quantitative estimates. To clarify, we have revised the manuscript and explicitly state that the trade-offs are described qualitatively.

In Abstract:

“The selection of both text preprocessing strategies and model architectures should be guided by note characteristics and practical considerations such as annotation burden.”

In Discussion:

“Finally, although we observed qualitative trade-offs between human annotation efforts (i.e., requirement for window vs. no requirement for split) and computational resources (e.g., runtime for BERT Short-Formers vs. Longformer), quantitative comparison was not feasible due to the lack of systematically recorded annotation time and computational costs.”

*Would reorganizing the background section to define all acronyms on first use improve clarity?*

**Response:** We thank the reviewer for this suggestion. We have revised the Background and Significance section to ensure that all acronyms are defined at first use to improve clarity.

*How generalizable do you expect the findings to be for drug classes beyond the two studied in the VUMC dataset?*

**Response:** We appreciate the reviewer’s insightful comment. Although the VUMC dataset focused on two drugs of interest, the models were also evaluated on the n2c2 dataset, which is substantially different. The n2c2 dataset includes inpatient discharge summaries from a predominantly adult population that are very long and covers adverse drug events (ADEs) for hundreds of medications, while the VUMC dataset was collected from an outpatient pediatric population with much shorter notes, focused on two drugs. The performance comparison between Clinical-Longformer over BERT Short-Formers across these datasets suggests the approach is likely generalizable to other drugs, as it is robust to variations in clinical contexts, documentation practices, medication coverage, and patient populations. However, we agree more extensive studies would be needed. We further detailed the n2c2 dataset and discussed this point as a limitation in the revised manuscript as follows:

In Materials and Methods:

“*The n2c2 data:* The n2c2 data was from the 2018 n2c2 shared task Track 2,(14,27–31) sourced from the MIMIC-III (Medical Information Mart for Intensive Care III) database.(32) It includes 505 discharge summaries from a predominantly adult population, annotated with drug names, ADEs, and their relationships, split into 303 training and 202 test notes. In the training set, 78.2% of notes contained ADEs, with 321 of approximately 2,220 (14.5%) unique drugs (not counting misspelled or inconsistent naming), and 864 of 16,223 (5.3%) drug mentions associated with at least one ADE. The test set showed a similar distribution, with 76.7% of notes containing ADEs, 241 of approximately 1,676 (14.4%) unique drugs, and 606 of 10,575 (5.7%) drug mentions associated with at least one ADE.”

In Discussion:

“Second, this study utilized the n2c2 dataset and a single institution-specific dataset. These two datasets are inherently distinct, reflecting substantially different clinical contexts, documentation practices, medication coverage, and patient populations, and thus provide meaningful heterogeneity that supports the assessment of model generalizability across varied healthcare settings. Despite that, validation across additional datasets, institutions, and drug classes remains warranted, particularly for medications with rare or heterogeneous ADE profiles.”

*Did you explore techniques like gradient accumulation to mitigate the GPU limitations affecting batch sizes?*

**Response:** We thank the reviewer for the comment. To address GPU memory limitations and increase the effective batch size, we used gradient accumulation (per-device batch size = 16, accumulation factor = 2) to reduce memory usage. Training was performed using mixed precision (fp16), along with gradient checkpointing and early stopping (min_delta = 0.001, patience = 2) to improve efficiency. These techniques enabled efficient training without compromising model performance. We have clarified this in the Materials and Methods section of the revised manuscript:

“BERT Short-Formers and Clinical-Longformer were fine-tuned using a per-device batch size of 16 with gradient accumulation of 2. Mixed precision training (fp16) and gradient checkpointing were employed to reduce memory usage and improve training efficiency. BERT Short-Formers were trained for 3 epochs with early stopping (patience = 2, minimum delta = 0.001) and default hyperparameters. Clinical-Longformer used dataset-specific attention windows based on text length, with n2c2 trained using default attention window of 512 and the VUMC dataset using a window size of 128 and a warmup ratio of 0.1. Training for Clinical-Longformer was conducted for 10 epochs, with all other hyperparameters consistent with BERT Short-Formers.”

*Were any notable differences in note structure/style observed between institutions that might affect model generalization?*

**Response:** We thank the reviewer for this comment. Although we did not perform a systematic comparison of note structure across institutions, we clarified in the revised manuscript that the two datasets used in this study differ substantially. The n2c2 dataset consists of longer inpatient discharge summaries derived from the MIMIC-III clinical database that covers ADEs for hundreds of medications in mostly adult patients. In contrast, the VUMC dataset contains shorter outpatient notes focused on two medications in pediatric patients. Evaluating the models across these datasets provides preliminary evidence of robustness to variation in clinical contexts, documentation practices, medication coverage, and patient populations. To address the comment, we have revised the manuscript as follows:

In Background and Significance:

“The two datasets contain markedly different types of clinical notes: the n2c2 data comprises fewer but longer notes, with a higher number of ADEs derived from hundreds of drug mentions, whereas the VUMC data includes more notes of shorter length, featuring more homogeneous ADEs focused on two drugs of interest.”

In Materials and Methods:

“*The n2c2 data:* The n2c2 data was from the 2018 n2c2 shared task Track 2,(14,27–31) sourced from the MIMIC-III (Medical Information Mart for Intensive Care III) database.(32) It includes 505 discharge summaries from a predominantly adult population, annotated with drug names, ADEs, and their relationships, split into 303 training and 202 test notes. In the training set, 78.2% of notes contained ADEs, with 321 of approximately 2,220 (14.5%) unique drugs (not counting misspelled or inconsistent naming), and 864 of 16,223 (5.3%) drug mentions associated with at least one ADE. The test set showed a similar distribution, with 76.7% of notes containing ADEs, 241 of approximately 1,676 (14.4%) unique drugs, and 606 of 10,575 (5.7%) drug mentions associated with at least one ADE.

*The VUMC data:* The VUMC data was extracted from Synthetic Derivative, de-identified EHRs at VUMC and was previously used in another study(33) that included a cohort of pediatric outpatients with ADEs related to two drugs of interest (citalopram/Celexa, escitalopram/Lexapro). As the gold standard labels were not available, we annotated the gold standards as described below. Our final cohort included 112 patients with a total of 1,541 notes that mentioned a drug of interest, split into: 1,109 notes in the training set, with 12.2% of notes containing ADEs, and 284 of 1,910 (14.9%) drug mentions being ADE-associated, and 432 notes in the test set, with 8.3% of notes containing ADE relations, and 59 of 757 (7.8%) drug mentions being ADE-associated.”

In Discussion:

“Second, this study utilized the n2c2 dataset and a single institution-specific dataset. These two datasets are inherently distinct, reflecting substantially different clinical contexts, documentation practices, medication coverage, and patient populations, and thus provide meaningful heterogeneity that supports the assessment of model generalizability across varied healthcare settings. Despite that, validation across additional datasets, institutions, and drug classes remains warranted, particularly for medications with rare or heterogeneous ADE profiles.”

**Reviewer #2:** The work presented in this paper explores an effective data processing method for extracting relevant information from clinical notes in EHRs for transformer-based models, ultimately aiming to develop an NLP system to detect ADEs in clinical notes. They investigated two data processing methods, window-based and split-based approaches, to find an optimal processing method.

*1. The authors report an F1-score of 0.832 and 0.868 on the n2c2 dataset using two methods. And a F1 socre of 0.720, 0.784 on the VUMC dataset. A more exhaustive comparison with the state-of-the-art is needed, specifically, compare to the University of California – San Francisco (UCSF)-BERT on the same two datasets.*

**Response:** We thank the reviewer for this suggestion. To explore the possibility of including University of California, San Francisco Bidirectional Encoder Representations from Transformer (UCSF-BERT) in our comparison, we contacted Dr. Vivek Rudrapatna, the principal investigator, to request access. We were informed that the model is not publicly available and requires execution of a Data Use Agreement (DUA) to obtain access.

While we appreciate the opportunity to request access, the DUA includes legal provisions that extend obligations beyond the project period (e.g., Section 5: “All of Recipient’s obligations herein shall survive the expiration or termination of this Agreement indefinitely.”). Given these requirements, we were unable to proceed with the agreement. We therefore focused our comparisons on publicly available BERT-based models, which facilitate reproducibility and broader use by the research community. We believe this provides a fair and practical comparison across the two datasets, which differ substantially in clinical contexts, documentation practices, medication coverage, and patient populations. To acknowledge the reviewer’s point, we have expanded the discussion about UCSF-BERT’s work in the revised manuscript and noted UCSF-BERT and other institution-specific models as potential directions for future comparative studies when access becomes available.

In Background and Significance:

“Furthermore, Silverman *et al.*(16) demonstrated that large language models (LLMs), such as University of California, San Francisco (UCSF)-BERT achieved higher accuracy in serious AE detection from clinical notes compared with previous methods.”

In Discussion:

“On institutional datasets, Silverman *et al.*(16) reported accuracy of 0.88-0.92 and macro F1 of 0.61-0.68 in drug-serious AE pair identification from annotated outpatient clinical notes at UCSF using Hierarchical UCSF BERT (H-UCSF-BERT). In comparison, our results on the VUMC dataset demonstrated improved performance with accuracy of 0.97 using Bert-base-uncased, and a macro F1 of 0.76 using Clinical-Longformer. It is important to note that their notes focused on 8 non-steroid immunosuppressants, whereas our study with the VUMC dataset focused specifically on AEs associated with two medications, citalopram and escitalopram. Although we also evaluated our approaches on the n2c2 dataset, which contains a broader set of drug-ADE relations, direct comparison was not possible because Silverman *et al.* did not report H-UCSF-BERT results on the n2c2 dataset.

…

Second, this study utilized the n2c2 dataset and a single institution-specific dataset. These two datasets are inherently distinct, reflecting substantially different clinical contexts, documentation practices, medication coverage, and patient populations, and thus provide meaningful heterogeneity that supports the assessment of model generalizability across varied healthcare settings. Despite that, validation across additional datasets, institutions, and drug classes remains warranted, particularly for medications with rare or heterogeneous ADE profiles. Institution-specific models such as UCSF-BERT, if made publicly available, represent promising avenues for future comparative studies, as they may offer further insight into model performance across diverse institutional note structures and patient populations, thereby strengthening the assessment of generalizability.”

*2. Please elaborate on the final class distribution of the VUMC dataset, after annotation of the VUMC Data. If imbalanced, what type of augmentation method/technique was used?*

**Response:** After annotation, the Vanderbilt University Medical Center (VUMC) dataset remained imbalanced: our final cohort included 112 patients with a total of 1,541 notes that mentioned a drug of interest, split into: 1,109 notes in the training set, with 12.2% of notes containing ADEs, and 284 of 1,910 (14.9%) drug mentions being ADE-associated, and 432 notes in the test set, with 8.3% of notes containing ADEs, and 59 of 757 (7.8%) drug mentions being ADE-associated. We did not perform data augmentation, as we aimed to evaluate model performance with minimal preprocessing and allow the models to learn directly from the original clinical text.

Instead, we used more comprehensive evaluation metrics and modeling strategies that are robust to imbalanced data. Specifically, we selected the area under the precision-recall curve (AUPRC) and macro F1 score as the primary metrics, supplemented with micro F1, macro precision, and macro recall, and applied per-fold threshold tuning during 5-fold cross-validation to account for rare ADEs. We also explored techniques such as class weighting and focal loss, but preliminary experiments with these approaches did not noticeably improve performance and introduced additional variability, so these were not implemented in the final analysis. Augmentation strategies may be a promising direction to explore, including paraphrasing, synthetic generation, and oversampling, to further improve the detection of rare ADEs. To address this comment, we have revised the manuscript as follows:

In Materials and Methods:

“Our final cohort included 112 patients with a total of 1,541 notes that mentioned a drug of interest, split into: 1,109 notes in the training set, with 12.2% of notes containing ADEs, and 284 of 1,910 (14.9%) drug mentions being ADE-associated, and 432 notes in the test set, with 8.3% of notes containing ADE relations, and 59 of 757 (7.8%) drug mentions being ADE-associated.

…

To address class imbalance, we explored mitigation strategies such as class weighting and focal loss. However, these approaches did not significantly improve performance and were therefore not implemented.

…

Given the extreme class imbalance in ADE cases, the area under the precision-recall curve (AUPRC) and macro F1 score were selected as the primary metrics, supplemented with micro F1 score, macro precision, and macro recall. Means and standard deviations (SDs) for all metrics on the test set are reported.”

In Conclusion:

“Moreover, data augmentation strategies such as paraphrasing, synthetic generation, oversampling, along with advanced long-document modeling techniques, including hybrid approaches that combine window- and split-based approaches, retrieval-augmented methods, and dual-pass architectures, could be explored to more effectively capture contextual information for ADE tasks.”

*3. From the following paragraph: “the gold standard label for “ADE positive” was created by combining AEpositive, AEpositiveNoJustification, and AEconditional labels, while the label for “ADE negative” was created by combining AEnegative and NoResponse labels”. This is not clear, there is no justification given for AEconditional going to the positive class; same thing, for the NoResponse going to the negative class. Please give you arguments for these decisions.*

**Response:** We could have limited annotation to the two final labels, AEpositive and AEnegative, from the outset. However, we intentionally adopted a more granular labeling scheme to capture nuances observed in the clinical notes. As a general principle, collecting detailed annotations upfront is preferable, as it is difficult to retrospectively reclassify coarse labels, whereas granular labels can be readily consolidated. Additionally, these finer-grained labels may support future development of other NLP tasks, such as extracting drug response information.

Accordingly, combining the labels was planned at the study design stage. The AEconditional label was grouped under “ADE positive” because it reflects cases where an ADE is conditionally present or implied, enabling the model to capture potential ADE signals. In contrast, the NoResponse label was included in the “ADE negative” because these instances indicate lack of drug efficacy rather than the presence of an ADE, which we verified during annotation. We have clarified this in the Materials and Methods section as follows:

“For our ADE classification task, the gold standard label for “ADE positive” was constructed by combining AEpositive, AEpositiveNoJustification, and AEconditional labels, while “ADE negative” combined AEnegative and NoResponse. AEconditional was categorized as positive because it indicates a conditionally present or implied ADE, thereby capturing potential signals. In contrast, NoResponse was categorized as negative as it reflects a lack of drug efficacy rather than the occurrence of an ADE, which was confirmed during annotation.”

*4. Figure 1 is of very low quality, please improve it.*

**Response:** To improve clarity and readability, we have revised Fig 1 by splitting it into two parts. While the diagram has been retained, we have moved the examples of text partitioning strategies to the Supporting Information to reduce visual clutter and enhance interpretability. In addition, we have regenerated the figure at higher resolution for the revised manuscript.

*5. The authors stated: “We evaluated the performance of each model by the average of three trials with different train-test split seeds for validation.” Authors should repeat all experiments using a well-known K-fold cross validation, using K = 5, or 10, depending on the final size of the dataset. Three trials are too few and without a proper rotation of the training/testing data folds.*

**Response:** In the revised manuscript, we implemented 5-fold cross-validation, with classification threshold tuning for each fold and evaluation on the hold-out test set. This approach ensures robust performance estimation with proper rotation of the training and validation data, while preserving the original test set for unbiased comparison with prior studies. The Materials and Methods section has been revised as follows:

“We used the original n2c2 train and test splits to allow direct comparison with prior studies. 5-fold cross-validation was performed on the training set for model development, using 4 folds for training and 1 for validation, with per-fold threshold tuning. The hold-out test set was reserved exclusively for final evaluation and was not used during training or tuning. The VUMC dataset followed the same protocol.”

*6. Results presented in Tables 1, 2, 3 and 4 should be the average values obtained from the K-fold cross validation suggested in the previous comment (5). Statistical tests are needed to claim a model outperforms another by a significant difference.*

**Response:** As suggested, we have updated Tables 1, 2, 3 and 4 to report the mean and standard deviation (SD) values across 5-fold cross-validation. Additionally, we applied the Wilcoxon signed-rank test to paired metrics to compare Clinical-Longformer (4-chunk setting) and PubMedBERT (10-chunk setting), which achieved the highest AUPRCs on the n2c2 dataset. Given the limited statistical power associated with a sample size of 5, we additionally conducted a 10-fold cross-validation as a sensitivity analysis, which yielded statistically significant results. However, considering the overall sample size of notes, we did not further pursue the 10-fold cross-validation and instead reported the mean and SD values across 5-fold cross-validation in the tables. The Results section has been revised accordingly to address this comment.

“We further conducted statistical testing using the Wilcoxon signed-rank test on paired results from 5-fold cross-validation to compare Clinical-Longformer (4-chunk setting) and PubMedBERT (10-chunk setting), which achieved the highest AUPRCs. The resulting p-values were 0.0625 for AUPRC, 0.0625 for micro F1, and 0.4375 for macro F1, indicating no statistically significant differences at the level of 0.05. However, the small number of folds (i.e., limited sample size) constrained the statistical power of these tests. Notably, for a paired sample size of 5 with no ties or zero differences, the minimum achievable p-value under a two-sided exact Wilcoxon signed-rank test is 0.0625. Thus, even if all paired AUPRC and macro F1 values for Clinical-Longformer exceeded those of PubMedBERT, statistical significance could not be reached. To further examine this limitation, we conducted additional analysis using 10-fold cross-validation. This yielded p-values of 0.0020 for AUPRC, 0.0020 for micro F1, and 0.1055 for macro F1. In this setting, 0.0020 represents the minimum possible p-value for a paired sample size of 10, consistent with all AUPRC values for Clinical-Longformer exceeding those of PubMedBERT. These findings suggested a potential performance difference between the models. However, we did not adopt 10-fold cross validation for all analyses, as it resulted in smaller validation sets, which may be suboptimal given the sample sizes. Therefore, we retained 5-fold cross-validation throughout and reported mean and SD values, without additional formal statistical testing due to the limited statistical power.”

*7. The authors claim that Clinical-Longformer significantly outperformed BERT Short-Formers. It is not possible to claim a model outperforms another without statistical tests, as suggested in 6).*

**Response:** Please see our response to Comment #6 above.

*8. Regarding one of the major limitations of transformer models on long text that exceeds the token limit, nowadays there are multiple practical strategies, both architectural and workflow-level, such as Retrieval-Augmented Generation (RAG), online transformers/chunkwise attention, sliding window attention, streaming transformers, dual-pass models (e.g., tiny windows but global memory tokens). Authors are highly recommended to expand their work from a fine-tuning technique to a combination of such techniques.*

**Response:** In this study, we addressed long text limitations primarily through model selection by using Clinical-Longformer along with practical data processing approach, which can natively handle longer sequences with an attention window. This allowed us to focus on systematic fine-tuning and controlled evaluation without introducing additional architectural or workflow-level complexity. To our knowledge, this is the first study to fine-tune a Longformer-based model for ADE detection, demonstrating its applicability to clinical NLP tasks. We acknowledge that alternative strategies such as RAG, streaming transformers, and dual-pass models may further enhance long document modeling. These approaches have been highlighted in the Conclusion section as important directions for future work.

“Moreover, data augmentation strategies such as paraphrasing, synthetic generation, oversampling, along with advanced long-document modeling techniques, including hybrid approaches that combine window- and split-based approaches, retrieval-augmented methods, and dual-pass architectures, could be explored to more effectively capture contextual information for ADE tasks.”

*9. It is good practice to ask corresponding authors questions about their work. For instance, in the work of Mahendran et al., they reported F-scores of 0.97 on Bert-base uncased and BioBERT, but it is not clear for the authors of this manuscript whether the scores are micro or macro F-scores for ADE-Drug detection. They assumed they are micro F-scores. In the case of the assumption being wrong, what is then the contribution of this paper?*

**Response:** We appreciate the comment. We contacted the corresponding author, Dr. Bridget T. McInnes, and confirmed that the reported F-score in Mahendran *et al.* is micro F1 (equivalent to accuracy in binary classification). Our study further contributes by providing a systematic comparison across multiple transformer models and datasets under consistent evaluation settings for ADE detection. We have clarified this in the revised manuscript.

“There are a few prior studies on the n2c2 dataset focused on ADE-Drug relation using BERT-based models. Our models achieved similar or improved performance (micro F1 score of 0.96 on Bert-base-uncased, 0.97 on BioClinicalBERT and BioBERT) compared to previously reported results by Wei *et al.* (micro F1 score of 0.80 on BERT-large-uncased and 0.81 on MIMIC BERT),(5,6,37) and Mahendran *et al.* (micro F1 scores of 0.97 on Bert-base-uncased, BioClinicalBERT and BioBERT).(3) In a recent work, Dai *et al.*(38) reported micro F1 scores (SDs) of 0.25, 0.52 (0.01), and 0.15 (0.22) on GPT-4, fine-tuned span-based models, and fine-tuned generative models, respectively, including generative artificial intelligence (AI) based approaches. Due to substantial class imbalance, we used AUPRC and macro metrics as the primary evaluation measures, as they better reflect performance on the minority class than micro F1 that is equivalent to accuracy in binary classification. Micro F1 scores were uniformly high and did not meaningfully differentiate model performance.

On institutional datasets, Silverman *et al.*(16) reported accuracy of 0.88-0.92 and macro F1 of 0.61-0.68 in drug-serious AE pair identification from annotated outpatient clinical notes at UCSF using Hierarchical UCSF BERT (H-UCSF-BERT). In comparison, our results on the VUMC dataset demonstrated improved performance with accuracy of 0.97 using Bert-base-uncased, and a macro F1 of 0.76 using Clinical-Longformer. It is important to note that their notes focused on 8 non-steroid immunosuppressants, whereas our study with the VUMC dataset focused specifically on AEs associated with two medications, citalopram and escitalopram. Although we also evaluated our approaches on the n2c2 dataset, which contains a broader set of drug-ADE relations, direct comparison was not possible because Silverman *et al.* did not report H-UCSF-BERT results on the n2c2 dataset.”

*10. This work focuses on ADEs associated with two drugs, specifically citalopram and escitalopram, using data obtained from a previous study investigating AEs for these drugs. Can your proposal generalize for other drugs? Please elaborate.*

**Response:** We thank the reviewer for the comment. While the VUMC dataset focused on two drugs of interest, the models were also evaluated on the n2c2 dataset, which differs substantially from the VUMC dataset. The n2c2 dataset consists of long inpatient discharge summaries covering ADEs for hundreds of medications; for instance, in its test set, 241 of approximately 1,676 drugs (not counting misspelled or inconsistent naming) are associated with at least one ADE. Performance across both datasets indicates that our approach is likely generalizable to other drugs, demonstrating robustness to variations in clinical contexts, documentation practices, medical coverage, and patient populations. Nevertheless, we acknowledge that further validation is needed to strengthen the generalizability of our findings. This point has been incorporated in the revised manuscript.

In Materials and Methods:

“*The n2c2 data:* The n2c2 data was from the 2018 n2c2 shared task Track 2,(14,27–31) sourced from the MIMIC-III (Medical Information Mart for Intensive Care III) database.(32) It includes 505 discharge summaries from a predominantly adult population, annotated with drug names, ADEs, and their relationships, split into 303 training and 202 test notes. In the training set, 78.2% of notes contained ADEs, with 321 of approximately 2,220 (14.5%) unique drugs (not counting misspelled or inconsistent naming), and 864 of 16,223 (5.3%) drug mentions associated with at least one ADE. The test set showed a similar distribution, with 76.7% of notes containing ADEs, 241 of approximately 1,676 (14.4%) unique drugs, and 606 of 10,575 (5.7%) drug mentions associated with at least one ADE.”

In Discussion:

“Second, this study utilized the n2c2 dataset and a single institution-specific dataset. These two datasets are inherently distinct, reflecting substantially different clinical contexts, documentation practices, medication coverage, and patient populations, and thus provide meaningful heterogeneity that supports the assessment of model generalizability across varied healthcare settings. Despite that, validation across additional datasets, institutions, and drug classes remains warranted, particularly for medications with rare or heterogeneous ADE profiles.”
