# Supplementary material for: Developing a natural language processing system using transformer-based models for adverse drug event detection in electronic health records
Source: PLoS One. 2026 Jul 1;21(7):e0350516. doi: 10.1371/journal.pone.0350516 (PMC13322497; doi:10.1371/journal.pone.0350516)
Supplement: Peer review history

# Peer review: Decision Letter 0

**DOI:** 10.1371/journal.pone.0350516.r001

**Authors:** Daniela Moctezuma

4 Dec 2025

PONE-D-25-22628

Developing a natural language processing system using transformer-based models for adverse drug event detection in electronic health records

PLOS One

Dear Dr. Choi,

Thank you for submitting your manuscript to PLOS ONE. After careful consideration, we feel that it has merit but does not fully meet PLOS ONE’s publication criteria as it currently stands. Therefore, we invite you to submit a revised version of the manuscript that addresses the points raised during the review process.

==============================

The comments of the reviewers ask for some important modifications and additional work to improve your manuscript, such as state-of-the-art additional comparison, deeper analysis of the results, as well as better image quality. So, I encourage the authors to consider all these suggestions and resubmit your work.

==============================

Please submit your revised manuscript by Jan 18 2026 11:59PM. If you will need more time than this to complete your revisions, please reply to this message or contact the journal office at plosone@plos.org. When you're ready to submit your revision, log on to https://www.editorialmanager.com/pone/ and select the 'Submissions Needing Revision' folder to locate your manuscript file.

Please include the following items when submitting your revised manuscript:

- A rebuttal letter that responds to each point raised by the academic editor and reviewer(s). You should upload this letter as a separate file labeled 'Response to Reviewers'.
- A marked-up copy of your manuscript that highlights changes made to the original version. You should upload this as a separate file labeled 'Revised Manuscript with Track Changes'.
- An unmarked version of your revised paper without tracked changes. You should upload this as a separate file labeled 'Manuscript'.

If you would like to make changes to your financial disclosure, please include your updated statement in your cover letter. Guidelines for resubmitting your figure files are available below the reviewer comments at the end of this letter.

If applicable, we recommend that you deposit your laboratory protocols in protocols.io to enhance the reproducibility of your results. Protocols.io assigns your protocol its own identifier (DOI) so that it can be cited independently in the future. For instructions see: https://journals.plos.org/plosone/s/submission-guidelines#loc-laboratory-protocols. Additionally, PLOS ONE offers an option for publishing peer-reviewed Lab Protocol articles, which describe protocols hosted on protocols.io. Read more information on sharing protocols at https://plos.org/protocols?utm_medium=editorial-email&utm_source=authorletters&utm_campaign=protocols.

We look forward to receiving your revised manuscript.

Kind regards,

Daniela Moctezuma

Academic Editor

PLOS One

Journal Requirements:

When submitting your revision, we need you to address these additional requirements.

1. Please ensure that your manuscript meets PLOS ONE's style requirements, including those for file naming. The PLOS ONE style templates can be found at https://journals.plos.org/plosone/s/file?id=wjVg/PLOSOne_formatting_sample_main_body.pdf and https://journals.plos.org/plosone/s/file?id=ba62/PLOSOne_formatting_sample_title_authors_affiliations.pdf

2. Please note that PLOS ONE has specific guidelines on code sharing for submissions in which author-generated code underpins the findings in the manuscript. In these cases, we expect all author-generated code to be made available without restrictions upon publication of the work. Please review our guidelines at https://journals.plos.org/plosone/s/materials-and-software-sharing#loc-sharing-code and ensure that your code is shared in a way that follows best practice and facilitates reproducibility and reuse.

3. We note that you have indicated that there are restrictions to data sharing for this study. For studies involving human research participant data or other sensitive data, we encourage authors to share de-identified or anonymized data. However, when data cannot be publicly shared for ethical reasons, we allow authors to make their data sets available upon request. For information on unacceptable data access restrictions, please see http://journals.plos.org/plosone/s/data-availability#loc-unacceptable-data-access-restrictions.

Before we proceed with your manuscript, please address the following prompts:

a) If there are ethical or legal restrictions on sharing a de-identified data set, please explain them in detail (e.g., data contain potentially identifying or sensitive patient information, data are owned by a third-party organization, etc.) and who has imposed them (e.g., a Research Ethics Committee or Institutional Review Board, etc.). Please also provide contact information for a data access committee, ethics committee, or other institutional body to which data requests may be sent.

b) If there are no restrictions, please upload the minimal anonymized data set necessary to replicate your study findings to a stable, public repository and provide us with the relevant URLs, DOIs, or accession numbers. Please see http://www.bmj.com/content/340/bmj.c181.long for guidelines on how to de-identify and prepare clinical data for publication. For a list of recommended repositories, please see https://journals.plos.org/plosone/s/recommended-repositories. You also have the option of uploading the data as Supporting Information files, but we would recommend depositing data directly to a data repository if possible.

Please update your Data Availability statement in the submission form accordingly.

4. In the online submission form, you indicated that your data is available only on request from a third party. Please note that your Data Availability Statement is currently missing [contact details for the third party, such as an email address or a link to where data requests can be made]. Please update your statement with the missing information.

5. We note that the grant information you provided in the ‘Funding Information’ and ‘Financial Disclosure’ sections do not match.

When you resubmit, please ensure that you provide the correct grant numbers for the awards you received for your study in the ‘Funding Information’ section.

6. Please amend your list of authors on the manuscript to ensure that each author is linked to an affiliation. Authors’ affiliations should reflect the institution where the work was done (if authors moved subsequently, you can also list the new affiliation stating “current affiliation:….” as necessary).

7. Please include captions for your Supporting Information files at the end of your manuscript, and update any in-text citations to match accordingly. Please see our Supporting Information guidelines for more information: http://journals.plos.org/plosone/s/supporting-information.

8. If the reviewer comments include a recommendation to cite specific previously published works, please review and evaluate these publications to determine whether they are relevant and should be cited. There is no requirement to cite these works unless the editor has indicated otherwise.

Additional Editor Comments :

The comments of the reviewers ask for some important modifications and additional work to improve your manuscript, such as state-of-the-art additional comparison, deeper analysis of the results, as well as better image quality. So, I encourage the authors to consider all these suggestions and resubmit your work.

[Note: HTML markup is below. Please do not edit.]

Reviewers' comments:

Reviewer's Responses to Questions

**Comments to the Author**

1. Is the manuscript technically sound, and do the data support the conclusions?

The manuscript must describe a technically sound piece of scientific research with data that supports the conclusions. Experiments must have been conducted rigorously, with appropriate controls, replication, and sample sizes. The conclusions must be drawn appropriately based on the data presented.

Reviewer #1: Yes

Reviewer #2: Partly

**********

2. Has the statistical analysis been performed appropriately and rigorously?

Reviewer #1: N/A

Reviewer #2: No

**********

3. Have the authors made all data underlying the findings in their manuscript fully available?

The PLOS Data policy requires authors to make all data underlying the findings described in their manuscript fully available without restriction, with rare exception (please refer to the Data Availability Statement in the manuscript PDF file). The data should be provided as part of the manuscript or its supporting information, or deposited to a public repository. For example, in addition to summary statistics, the data points behind means, medians and variance measures should be available. If there are restrictions on publicly sharing data—e.g. participant privacy or use of data from a third party—those must be specified.

Reviewer #1: No

Reviewer #2: Yes

**********

4. Is the manuscript presented in an intelligible fashion and written in standard English?

PLOS ONE does not copyedit accepted manuscripts, so the language in submitted articles must be clear, correct, and unambiguous. Any typographical or grammatical errors should be corrected at revision, so please note any specific errors here.

Reviewer #1: Yes

Reviewer #2: Yes

**********

5. Review Comments to the Author

Please use the space provided to explain your answers to the questions above. You may also include additional comments for the author, including concerns about dual publication, research ethics, or publication ethics. (Please upload your review as an attachment if it exceeds 20,000 characters)

Reviewer #1: Did you consider hybrid approaches that might combine advantages of both window-based and split-based processing methods?

Could you elaborate on the specific patterns of instability observed with Clinical-Longformer's non-default attention windows (e.g., types of performance drops or convergence failures)?

Were there particular label categories in the annotation process that showed lower agreement than the overall Kappa of 0.820, and how were these resolved?

Did you perform statistical tests to determine if the performance differences between models (e.g., Clinical-Longformer's 0.868 vs PubMedBERT's 0.832 F-score) were significant?

What specific measures were taken to ensure the evaluation wasn't skewed by the extreme class imbalance in the VUMC dataset (8.33-12.17% ADE-positive)?

What range of variance was observed in performance metrics across the multiple trials with different seeds?

Could you provide more details about the VUMC data request process, including approval timelines and required documentation?

Have you considered creating and sharing a synthetic version of the VUMC dataset that preserves clinical characteristics but removes PHI?

Did you use the original n2c2 train/test splits from the shared task or create new splits?

Would consolidating model configuration details into a dedicated "Model Specifications" subsection improve readability?

Could you add specific metrics (e.g., annotation time, compute hours) to quantify the "allocation trade-offs of human and computational power" mentioned in the abstract?

Would reorganizing the background section to define all acronyms on first use improve clarity?

How generalizable do you expect the findings to be for drug classes beyond the two studied in the VUMC dataset?

Did you explore techniques like gradient accumulation to mitigate the GPU limitations affecting batch sizes?

Were any notable differences in note structure/style observed between institutions that might affect model generalization?

Reviewer #2: The work presented in this paper explores an effective data processing method for extracting relevant information from clinical notes in EHRs for transformer-based models, ultimately aiming to develop an NLP system to detect ADEs in clinical notes. They investigated two data processing methods, window-based and split-based approaches, to find an optimal processing method.

1. The authors report an F1-score of 0.832 and 0.868 on the n2c2 dataset using two methods. And a F1 socre of 0.720, 0.784 on the VUMC dataset. A more exhaustive comparison with the state-of-the-art is needed, specifically, compare to the University of California – San Francisco (UCSF)-BERT on the same two datasets.

2. Please elaborate on the final class distribution of the VUMC dataset, after annotation of the VUMC Data. If imbalanced, what type of augmentation method/technique was used?

3. From the following paragraph: “the gold standard label for “ADE positive” was created by combining AEpositive, AEpositiveNoJustification, and AEconditional labels, while the label for “ADE negative” was created by combining AEnegative and NoResponse labels”. This is not clear, there is no justification given for AEconditional going to the positive class; same thing, for the NoResponse going to the negative class. Please give you arguments for these decisions.

4. Figure 1 is of very low quality, please improve it.

5. The authors stated: “We evaluated the performance of each model by the average of three trials with different train-test split seeds for validation.” Authors should repeat all experiments using a well-known K-fold cross validation, using K = 5, or 10, depending on the final size of the dataset. Three trials are too few and without a proper rotation of the training/testing data folds.

6. Results presented in Tables 1, 2, 3 and 4 should be the average values obtained from the K-fold cross validation suggested in the previous comment (5). Statistical tests are needed to claim a model outperforms another by a significant difference.

7. The authors claim that Clinical-Longformer significantly outperformed BERT Short-Formers. It is not possible to claim a model outperforms another without statistical tests, as suggested in 6).

8. Regarding one of the major limitations of transformer models on long text that exceeds the token limit, nowadays there are multiple practical strategies, both architectural and workflow-level, such as Retrieval-Augmented Generation (RAG), online transformers/chunkwise attention, sliding window attention, streaming transformers, dual-pass models (e.g., tiny windows but global memory tokens). Authors are highly recommended to expand their work from a fine-tuning technique to a combination of such techniques.

9. It is good practice to ask corresponding authors questions about their work. For instance, in the work of Mahendran et al., they reported F-scores of 0.97 on Bert-base uncased and BioBERT, but it is not clear for the authors of this manuscript whether the scores are micro or macro F-scores for ADE-Drug detection. They assumed they are micro F-scores. In the case of the assumption being wrong, what is then the contribution of this paper?

10. This work focuses on ADEs associated with two drugs, specifically citalopram and escitalopram, using data obtained from a previous study investigating AEs for these drugs. Can your proposal generalize for other drugs? Please elaborate.

**********

6. PLOS authors have the option to publish the peer review history of their article (what does this mean?). If published, this will include your full peer review and any attached files.

If you choose “no”, your identity will remain anonymous but your review may still be made public.

**Do you want your identity to be public for this peer review?**  For information about this choice, including consent withdrawal, please see our Privacy Policy.

Reviewer #1: No

Reviewer #2: No

**********

[NOTE: If reviewer comments were submitted as an attachment file, they will be attached to this email and accessible via the submission site. Please log into your account, locate the manuscript record, and check for the action link "View Attachments". If this link does not appear, there are no attachment files.]

To ensure your figures meet our technical requirements, please review our figure guidelines: https://journals.plos.org/plosone/s/figures

You may also use PLOS’s free figure tool, NAAS, to help you prepare publication quality figures: https://journals.plos.org/plosone/s/figures#loc-tools-for-figure-preparation.

NAAS will assess whether your figures meet our technical requirements by comparing each figure against our figure specifications.

# Author comment: Author response to Decision Letter 1

**DOI:** 10.1371/journal.pone.0350516.r002

15 Apr 2026

We uploaded our response to the reviewers' comments as a separate document.

Attachment Submitted filename: AEproject_Response_to_Reviewers_0411.docx

# Peer review: Decision Letter 1

**DOI:** 10.1371/journal.pone.0350516.r003

**Authors:** Daniela Moctezuma, Daniela Moctezuma

15 May 2026

Developing a natural language processing system using transformer-based models for adverse drug event detection in electronic health records

PONE-D-25-22628R1

Dear Dr. Choi,

We’re pleased to inform you that your manuscript has been judged scientifically suitable for publication and will be formally accepted for publication once it meets all outstanding technical requirements.

Within one week, you’ll receive an e-mail detailing the required amendments. When these have been addressed, you’ll receive a formal acceptance letter and your manuscript will be scheduled for publication.

An invoice will be generated when your article is formally accepted. Please note, if your institution has a publishing partnership with PLOS and your article meets the relevant criteria, all or part of your publication costs will be covered. Please make sure your user information is up-to-date by logging into Editorial Manager at Editorial Manager® and clicking the ‘Update My Information' link at the top of the page. For questions related to billing, please contact billing support.

If your institution or institutions have a press office, please notify them about your upcoming paper to help maximize its impact. If they’ll be preparing press materials, please inform our press team as soon as possible -- no later than 48 hours after receiving the formal acceptance. Your manuscript will remain under strict press embargo until 2 pm Eastern Time on the date of publication. For more information, please contact onepress@plos.org.

Kind regards,

Daniela Moctezuma

Academic Editor

PLOS One

Additional Editor Comments (optional):

Reviewers' comments:

Reviewer's Responses to Questions

**Comments to the Author**

1. If the authors have adequately addressed your comments raised in a previous round of review and you feel that this manuscript is now acceptable for publication, you may indicate that here to bypass the “Comments to the Author” section, enter your conflict of interest statement in the “Confidential to Editor” section, and submit your "Accept" recommendation.

Reviewer #2: All comments have been addressed

**********

2. Is the manuscript technically sound, and do the data support the conclusions?

The manuscript must describe a technically sound piece of scientific research with data that supports the conclusions. Experiments must have been conducted rigorously, with appropriate controls, replication, and sample sizes. The conclusions must be drawn appropriately based on the data presented.

Reviewer #2: Yes

**********

3. Has the statistical analysis been performed appropriately and rigorously?

Reviewer #2: Yes

**********

4. Have the authors made all data underlying the findings in their manuscript fully available?

The PLOS Data policy requires authors to make all data underlying the findings described in their manuscript fully available without restriction, with rare exception (please refer to the Data Availability Statement in the manuscript PDF file). The data should be provided as part of the manuscript or its supporting information, or deposited to a public repository. For example, in addition to summary statistics, the data points behind means, medians and variance measures should be available. If there are restrictions on publicly sharing data—e.g. participant privacy or use of data from a third party—those must be specified.

Reviewer #2: No

**********

5. Is the manuscript presented in an intelligible fashion and written in standard English?

PLOS ONE does not copyedit accepted manuscripts, so the language in submitted articles must be clear, correct, and unambiguous. Any typographical or grammatical errors should be corrected at revision, so please note any specific errors here.

Reviewer #2: Yes

**********

6. Review Comments to the Author

Please use the space provided to explain your answers to the questions above. You may also include additional comments for the author, including concerns about dual publication, research ethics, or publication ethics. (Please upload your review as an attachment if it exceeds 20,000 characters)

Reviewer #2: Most comments and concerns from reviewers have been addressed satisfactorily. Suggestions not implemented have been fully justified by detail explanations and clarifications.

**********

7. PLOS authors have the option to publish the peer review history of their article (what does this mean?). If published, this will include your full peer review and any attached files.

If you choose “no”, your identity will remain anonymous but your review may still be made public.

**Do you want your identity to be public for this peer review?**  For information about this choice, including consent withdrawal, please see our Privacy Policy.

Reviewer #2: No

**********

# Editor report: Acceptance letter

**DOI:** 10.1371/journal.pone.0350516.r004

**Authors:** Daniela Moctezuma, Daniela Moctezuma

PONE-D-25-22628R1

PLOS One

Dear Dr. Choi,

I'm pleased to inform you that your manuscript has been deemed suitable for publication in PLOS One. Congratulations! Your manuscript is now being handed over to our production team.

At this stage, our production department will prepare your paper for publication. This includes ensuring the following:

* All references, tables, and figures are properly cited

* All relevant supporting information is included in the manuscript submission,

* There are no issues that prevent the paper from being properly typeset

You will receive further instructions from the production team, including instructions on how to review your proof when it is ready. Please keep in mind that we are working through a large volume of accepted articles, so please give us a few days to review your paper and let you know the next and final steps.

Lastly, if your institution or institutions have a press office, please let them know about your upcoming paper now to help maximize its impact. If they'll be preparing press materials, please inform our press team within the next 48 hours. Your manuscript will remain under strict press embargo until 2 pm Eastern Time on the date of publication. For more information, please contact onepress@plos.org.

You will receive an invoice from PLOS for your publication fee after your manuscript has reached the completed accept phase. If you receive an email requesting payment before acceptance or for any other service, this may be a phishing scheme. Learn how to identify phishing emails and protect your accounts at https://explore.plos.org/phishing.

If we can help with anything else, please email us at customercare@plos.org.

Thank you for submitting your work to PLOS ONE and supporting open access.

Kind regards,

PLOS ONE Editorial Office Staff

on behalf of

Dr. Daniela Moctezuma

Academic Editor

PLOS One
